# Comparing Triflate and Hexafluorophosphate Anions of Ionic Liquids in Polymer Electrolytes for Supercapacitor Applications

**DOI:** 10.3390/ma7054019

**Published:** 2014-05-21

**Authors:** Chiam-Wen Liew, S. Ramesh

**Affiliations:** Centre for Ionics University of Malaya, Department of Physics, Faculty of Science, University of Malaya, Lembah Pantai, 50603 Kuala Lumpur, Malaysia; E-Mail: liewchiamwen85@gmail.com

**Keywords:** corn starch, ionic liquid, triflate anion, hexafluorophosphate anion, supercapacitor

## Abstract

Two different ionic liquid-based biopolymer electrolyte systems were prepared using a solution casting technique. Corn starch and lithium hexafluorophosphate (LiPF_6_) were employed as polymer and salt, respectively. Additionally, two different counteranions of ionic liquids, viz. 1-butyl-3-methylimidazolium hexafluorophosphate (BmImPF_6_) and 1-butyl-3-methylimidazolium trifluoromethanesulfonate (also known as 1-butyl-3-methylimidazolium triflate) (BmImTf) were used and studied in this present work. The maximum ionic conductivities of (1.47 ± 0.02) × 10^−4^ and (3.21 ± 0.01) × 10^−4^ S·cm^−1^ were achieved with adulteration of 50 wt% of BmImPF_6_ and 80 wt% of BmImTf, respectively at ambient temperature. Activated carbon-based electrodes were prepared and used in supercapacitor fabrication. Supercapacitors were then assembled using the most conducting polymer electrolyte from each system. The electrochemical properties of the supercapacitors were then analyzed. The supercapacitor containing the triflate-based biopolymer electrolyte depicted a higher specific capacitance with a wider electrochemical stability window compared to that of the hexafluorophosphate system.

## 1. Introduction

A supercapacitor (also known as an electrochemical capacitor or ultracapacitor) is a promising candidate for an energy storage device. A supercapacitor can be projected as a future power source for high power applications such as hybrid-power systems for electric vehicles and military devices, utility load maintenance, heavy-load starting assists for diesel locomotives, and medical applications. Apart from that, it offers lower power applications, for example, the camera, flash equipment, lasers, pulsed-light generators and memory back-up systems in electronic equipment [1–4]. A supercapacitor possesses several advantages over a secondary rechargable battery such as long cycle life, high power density, short charging time, rapid energy delivery as well as being environmentally friendly [4,5]. Compared with the conventional dielectric capacitor, it has a higher energy density due to the large surface area of the electrode materials [4,6]. The capacitive behavior of a supercapacitor is based on the ability to form an electrical double layer at the polarizable electrode-electrolyte interface which arises from the charge separation between the high specific area carbon and an organic electrolyte [6,7].

Starch is a potential candidate as a host polymer due to its unique characteristics such as being inexpensive, abundant in nature, having good compatibility, renewable, excellent solubility and superior mechanical strength [8–10]. Lithium hexafluorophosphate (LiPF_6_) which acts as a charge carrier provider is used in this present work. Even though a similar project was reported in our previous publication [11,12], none of the application was applied using the same polymer electrolyte system. This current work will focus on the electrochemical properties of the fabricated supercapacitor using two symmetrical activated carbon-based electrodes. Apart from that, two different counteranions of ionic liquids (hexafluorophosphate, PF_6_^−^ and triflate, Tf^−^) are employed and compared in this study. The main objective of this work is to investigate the effect of the anion of the ionic liquid on the biopolymer electrolytes in the supercapacitor application.

## 2. Results and Discussion

### 2.1. Ambient Temperature-Ionic Conductivity Studies

**Figure 1.**
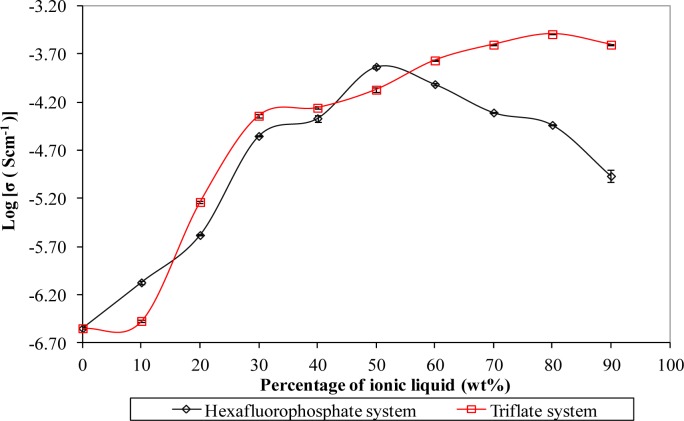
Ionic conductivity of ionic liquid-based biopolymer electrolytes with respect to the mass fraction of ionic liquid.

Figure 1 depicts the ionic conductivity of biopolymer electrolytes for both systems. The ionic conductivity of biopolymer electrolytes is found to increase with the mass fraction of the ionic liquid, up to a maximum level. The enhancement of ionic conductivity is due to the strong plasticizing effect of the ionic liquid, as reported in our study [11]. The plasticizing effect tends to weaken the coordination bonds within the macromolecules through the interaction between BmIm cations of the ionic liquid with the negatively charged oxygen of corn starch. As a result, the cations of the ionic liquids would favor a reduction in the solvation of the charge carriers (refer to lithium cations in this work) by the polymer matrix. Therefore, the charge carriers can be decoupled and transported along the polymer backbone easily which is in accordance with higher ionic conductivity [11]. In order to investigate the effect of the counteranions of the ionic liquid on the ionic hopping mechanism process, ionic liquids bearing different type of anions (hexafluorophosphate and triflate) were used in this present work where their common countercations are 1-butyl-3-methylimidazolium. Hexafluorophosphate and triflate anions are well known as “weakly coordinating” or “non-coordinating” anions as reported by Hayashida *et al.* [13]. This inherent behavior helps ionic dissociation which improves the conduction process. Therefore, hexafluorophosphate and triflate-based ionic liquids have been chosen as additives to increase the ionic conductivity in the development of polymer electrolytes.

Upon addition of 50 wt% of BmImPF_6_, ionic conductivity of (1.47 ± 0.02) × 10^−4^ S·cm^−1^ was achieved at room temperature as reported in our previous published paper [11]. However, the highest ambient temperature-ionic conductivity of triflate based-biopolymer electrolytes is increased to (3.21 ± 0.01) × 10^−4^ S·cm^−1^ with inclusion of 80 wt% of BmImTf.

This is strongly correlated to the effect of counteranion in the ionic liquid because the host polymer and conducting salt used are the same in both studies. We suggest that the increment of ionic conductivity is due to the bigger size of triflate anions. According to the literature, the volume of triflate anions is around 86.9 Ǻ^3^, whereas for the hexafluorophosphate anions, it is around 73 Ǻ^3^ [14]. The bigger size of the triflate anion could enhance the self-dissociating property. In other words, the triflate anion can be dissociated easily compared to the hexafluorophosphate anion due to its bulky size. So, more and more cations can be detached easily from the transient bonding with anions. As previously mentioned, the cations of the ionic liquid could soften the polymer backbone through interactive bonding. Therefore, the polymer chains become more flexible, producing more conducting pathways which assist the ionic transportation within the macromolecules.

**Figure 2.**
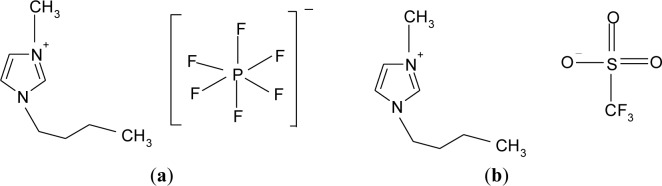
Structures of (**a**) 1-butyl-3-methylimidazolium hexafluorophosphate (BmImPF_6_); and (**b**) 1-butyl-3-methylimidazolium trifluoromethanesulfonate (BmImTf).

In addition, from a chemistry viewpoint, the triflate anion is a good leaving group compared to hexafluorophosphate anion due to its strong electron-withdrawing groups. The phosphorus is only surrounded by six fluorides in the hexafluorophosphate anion as shown in Figure 2a. On the other hand, triflate anions are bound by a sulfonate (SO_3_) and a perfluoromethyl group (CF_3_) as illustrated in Figure 2b.

**Figure 3.**
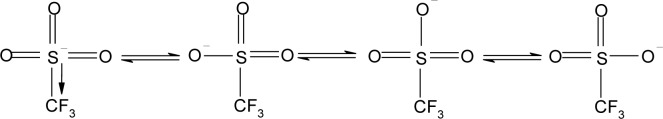
Resonance state of triflate anions.

Moreover, the triflate anion has a strong structure dissociation tendency as reported by Mitra *et al.* [1]. Again, this labile ligand can enhance the ion dissociation into a compound which enriches the softening in the polymer backbone. Apart from that, the delocalization process of triflate anions is a way of enhancing the ionic conductivity. The triflate anion can be delocalized into four resonances to form a stable product due to the inductive effect between the electron withdrawing groups and its conjugated structure, as illustrated in Figure 3 [15].

However, the hexafluorophosphate anion does not show any resonance structure. The delocalization of electrons would result in stabilization onto the anions and reduce the tendency of coordination with the bulky cations [16]. Therefore, the triflate anion can be dissociated easily in comparison to the hexafluorophosphate anion. The ease of this ion dissociation improves ionic migration within the polymer chains and thus leads to higher ionic conductivity.

### 2.2. Linear Sweep Voltammetry (LSV)

**Figure 4.**
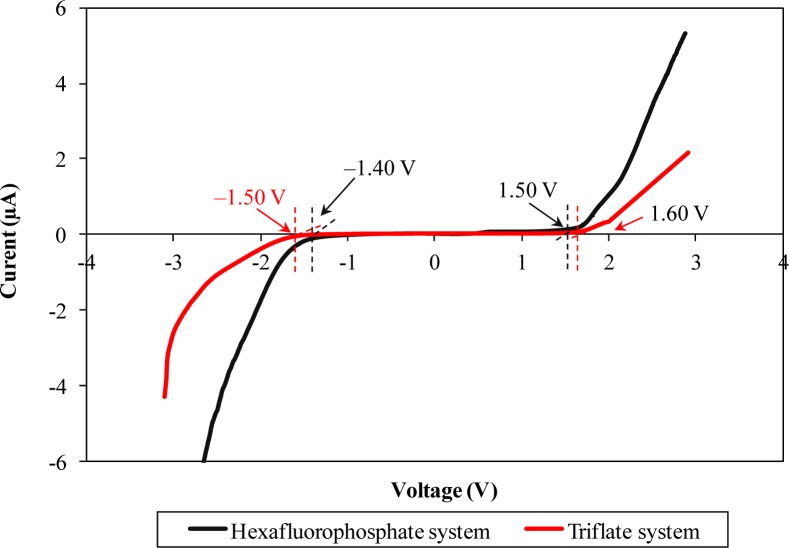
Linear sweep voltammetry (LSV) responses of different ionic liquid-based biopolymer electrolyte systems.

LSV responses of two different types of ionic liquid-based biopolymer electrolytes are illustrated in Figure 4. Hexafluorophosphate-based biopolymer electrolyte can be operated up to 2.90 V, from −1.40 to 1.50 V. On the other hand, triflate-based biopolymer electrolyte portrays wider electrochemical stability of 3.1 V, in the potential range from −1.50 to 1.60 V.

The wider electrochemical stability range infers that the triflate-based ionic liquid has a better electrochemical stability feature compared to the hexafluorophostate-based ionic liquid. This feature is most probably attributable to electron delocalization of triflate anions which is induced by the strong electron withdrawing group. The triflate anions will undergo an electron delocalization process to form a stable state, as mentioned in Section 3.1. Since the triflate anions form stable resonance structures, the electrochemical stability would be improved as expected. Beyond the potential range, the polymer electrolyte starts to be degraded.

### 2.3. Cyclic Voltammetry (CV)

**Figure 5.**
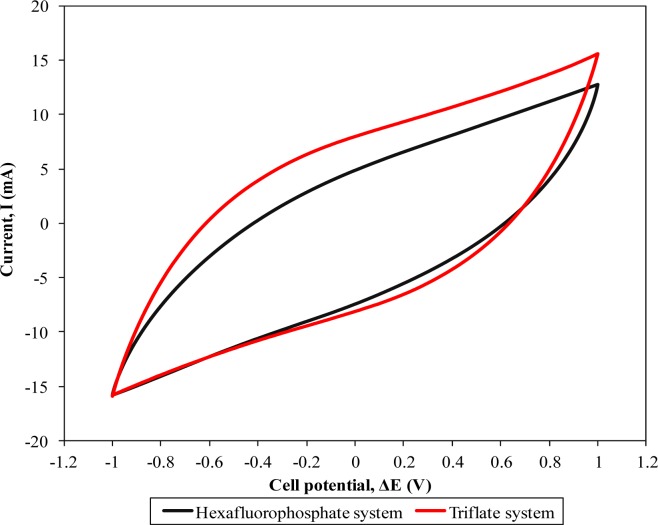
Cyclic voltammograms of different ionic liquid-based biopolymer electrolyte systems.

Figure 5 depicts the evaluation of cyclic voltammetries of two different supercapacitors. Neither of these curves show rectangular shapes. However, the voltammetry approaching the ideal shape was observed in both CV curves.

This feature denotes the capacitive behavior of the supercapacitors [17]. Redox peaks are absent in the figure indicating a non-faradic process in the supercapacitors. This non-faradic feature infers the formation of an electrical double layer which arises from ion absorption at the electrode-electrolyte interface. So, the contribution from electrons is negligible in this present work. The capacitance of the assembled supercapacitors was thus determined. The supercapacitor containing the highest conducting hexafluorophosphate-based polymer electrolyte illustrates a specific capacitance of 36.79 Fg^−1^. On the other hand, higher specific capacitance around 42.44 Fg^−1^ is attained for the triflate-based polymer system.

Higher ionic conductivity of the polymer electrolyte is the main attributor of the increase in specific capacitance of the supercapacitor, owing to the strong plasticizing effect of the ionic liquid. Since a higher ionic conductivity of the polymer electrolytes infers higher ionic transportation, the ions can migrate easily from one terminal to another of the electrodes promoting the formation of an electrical double layer at the electrode-electrolyte boundary. The supercapacitor containing triflate-based polymer system exhibits higher specific capacitance as shown in Figure 5. The higher capacitive behavior is also promoted by the higher ionic liquid concentration in the triflate-based polymer electrolytes. Doping of ionic liquid could produce sticky-like biopolymer electrolytes. The adhesive behavior of biopolymer electrolytes becomes more apparent when higher ionic liquid mass loading is added. As a result, this adhesion improves the interfacial contact between electrode-electrolyte. Therefore, the charge carriers can be transported easily. Hence, this ease of ionic hopping could improve the formation of an electrical double layer in the supercapacitor and thus increase the energy storage performance in the supercapacitor.

### 2.4. Electrochemical Impedance Spectroscopy (EIS)

The EIS of the fabricated cells also studied and demonstrated as below.

**Figure 6.**
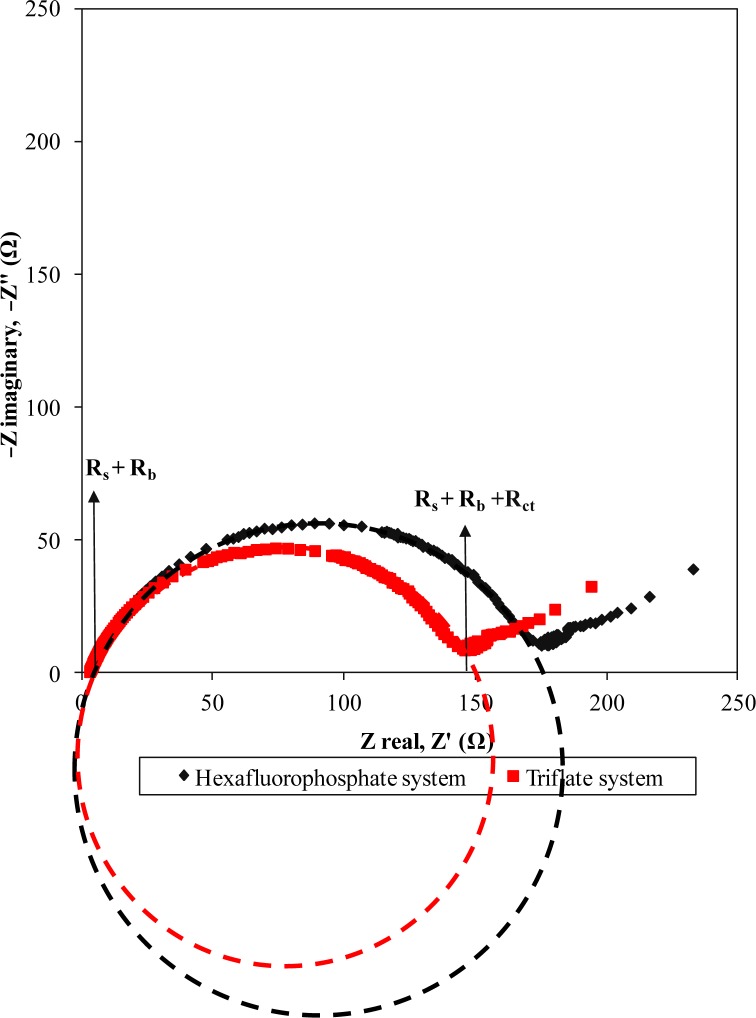
Nquist plots of EDLCs using both systems.

The Nquist plot (see Figure 6) is generally divided into two regions that are a semicircle at high frequency and a spike at low frequency. The plots do not start at the origin. This observation implies the presence of resistances consisting of series resistance (*R_s_*) and bulk resistance (*R_b_*).This resistance originates from the bulk resistance (*R_b_*) of the polymer electrolyte, series resistance (*R_s_*) of the connector and internal resistance of the electrode for ion diffusion as well as ohmic loss. Both systems show almost similar resistance at the high frequency end. The intercept of the semicircle and spike gives rise to the combination resistances of *R_s_*, *R_b_* and the charge transfer resistance (*R_ct_*). The effect of the counteranion in the ionic liquid is also observed in EIS plots. We note that the *R_ct_* of the EDLC comprised of the triflate system is relatively lower than that of the hexafluorophosphate system. So, we can conclude that the lower charge transfer resistance of the triflate-based EDLC contributes to the higher capacitance value compared to the hexafluorophosphate system. The bigger size of the anion could help in ion dissociation and hence reduce the barrier that the charge carriers have to overcome for transportation. As a result, the charge carriers in the triflate-based biopolymer electrolytes can migrate more easily than in the hexafluorophosphate system. On the other hand, the spike denotes the formation of a double layer at the electrolyte-electrode interface. Hexafluorophosphate and triflate-based EDLCs show a specific capacitance of 36.75 Fg^−1^ and 44.09 Fg^−1^, respectively. The result is comparable with the CV findings.

### 2.5. Galvanostatic Charge-Discharge Performance (GCD)

**Figure 7.**
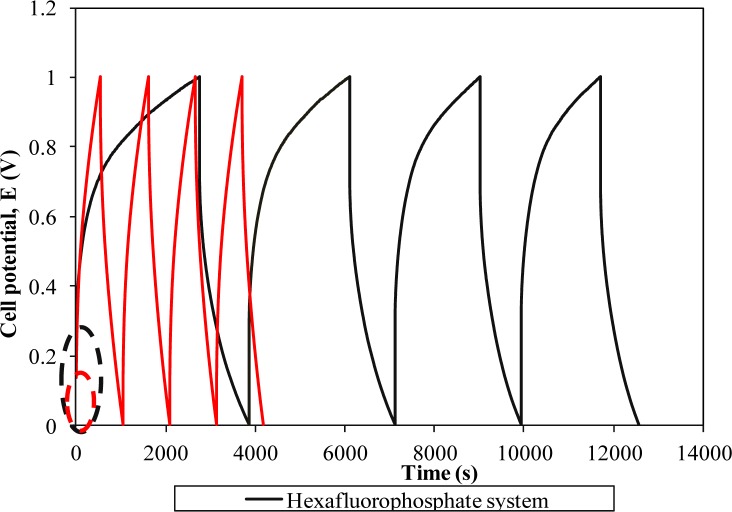
Galvanostatic charge-discharge performances of different ionic liquid-based biopolymer electrolyte systems.

Figure 7 represents the galvanostatic charge-discharge curves of two different systems.

The linear charge-discharge behavior in both curves divulges the non-redox reaction in the supercapacitor. Therefore, the energy storage mechanism in this supercapacitor is based on the ion accumulation at the electrode-electrolyte interface. A triflate-based supercapacitor portrays a more symmetrical behavior in comparison to the hexafluorophosphate system revealing superior capacitive properties [18]. Apart from good capacity, this result indicates higher Coulombic efficiency of the supercapacitor as the charging and discharging times are almost the same. The starting potential of both cells in the charging process deviates from 0 V. This initial change in potential in the charging process is mainly attributed to the internal resistance (known as ohmic loss) in the cells. Charge transfer resistance, bulk resistance of polymer electrolyte and depletion of polymer electrolyte might impede the ion absorption onto the electrodes and hence boost the internal resistance of the cells [1,2,7]. Another important phenomenon is also observed when we compare two charge-discharge curves. The hexafluorophosphate system requires a higher potential to be charged than the triflate system. The triflate system shows the initial charging potential at 0.14 V. In contrast, the starting potential for the hexafluorophosphate system has been doubled up, to around 0.28 V. This observation reflects higher internal resistance of the cell comprising the hexafluorophosphate system which inhibits the charge accumulation at the boundary. As a result, this could lessen the capacitive behavior of the supercapacitor. Poor interfacial contact of the hexafluorophosphate system might be the main reason for the high internal resistance of the supercapacitor. The specific capacitance of both cells is also determined using this technique.The triflate system-based supercapacitor exemplifies specific capacitance of 41.75 Fg^−1^, whereas the hexafluorophosphate system demonstrates a lower capacitance value, around 37.07 Fg^−1^. The obtained result is approximately the same as reported in the CV study. Lower capacitance of the hexafluorophosphate system is primarily due to the lower ionic conductivity of the polymer electrolyte, as explained in Section 3.5.

**Figure 8.**
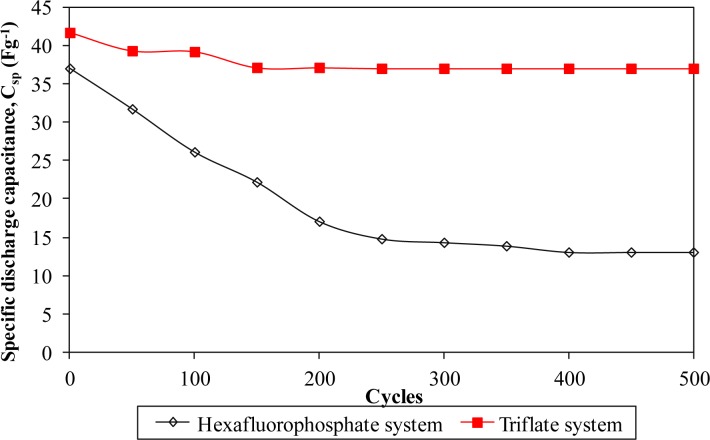
Specific capacitance of different ionic liquid-based biopolymer electrolyte systems over 500 cycles.

**Figure 11.**
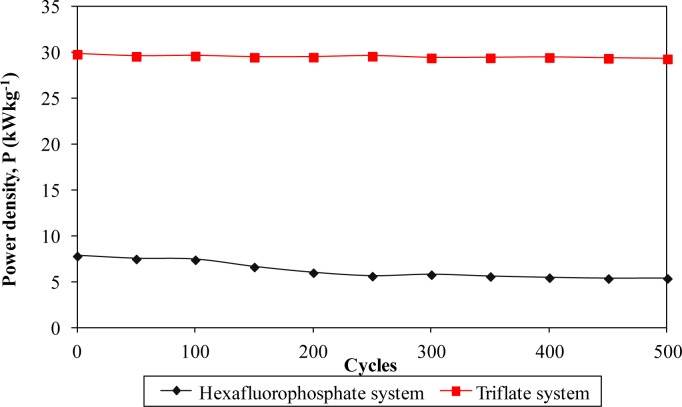
Power density of different ionic liquid-based biopolymer electrolyte systems over 500 cycles.

Furthermore, the electrochemical performance in terms of stability is investigated by charging and discharging the cells for 500 cycles. Figures 8 and 11 describe the specific capacitance, Coulombic efficiency, energy density and power density of both types of supercapacitors over 500 cycles, respectively. The specific capacitance and energy density of both cells decrease abruptly with increasing cycle number.

On the other hand, the power density of both cells reduces gradually as the charging and discharging cycle increase. Such reduction might be ascribed to the depletion of the polymer electrolytes and immobilization of the charge carriers after prolonged charge and discharge processes. However, the Coulombic efficiency of both cells increases with the cycle number. The triflate system shows an efficiency above 90% within 500 cycles, whereas the hexafluorophosphate system reveals lower efficiency. Based on the results, the hexafluorophosphate system illustrates rapid decreases in specific capacitance, energy density and power density from the initial to the 400th cycle. This means that the supercapacitor containing the hexafluorophosphate-based ionic liquid can only be stable after 400 charging and discharging cycles. However, triflate-based supercapacitor displays have improved electrochemical stability, where the specific capacitance, energy density and power density remain unchanged after 300 cycles. We suggest that the wider electrochemical stability is related to the delocalization process of triflate anions which helps in forming stable anions. The triflate-based supercapacitor exhibits better electrochemical properties than that of the hexafluorophosphate-based system as higher specific capacitance, higher Coulombic efficiency, higher energy density and higher power density are obtained in Figures 8 and 11. This illustration is strongly related to the higher ionic conductivity of the biopolymer electrolytes. Based on the findings, it can be concluded that the triflate-based supercapacitor is a promising candidate compared to the hexafluorophosphate system as it exhibits superb electrochemical properties.

## 3. Experimental Section

### 3.1. Materials

Biopolymer electrolytes were prepared by a solution casting technique. Biodegradable corn starch and LiPF_6_ were employed as host polymer and salt, respectively. On the other hand, different counteranions of ionic liquids, *i.e.*, 1-butyl-3-methylimidazolium hexafluorophosphate (BmImPF_6_) (purchased from Sigma-Alridch with purity of ≥95%, St. Louis, MO, USA) or 1-butyl-3-methylimidazolium trifluoromethanesulfonate (BmImTf) (purchased from Sigma-Alridch with purity of ≥98.5%) were used in this present work. The structures of hexafluorophosphate and triflate based ionic liquids are shown in Figure 2. All the materials were used as received.

### 3.2. Preparation of Biopolymer Electrolytes

The ratio of corn starch to LiPF_6_ is fixed as 80 wt% to 20 wt%. Appropriate amounts of corn starch and LiPF_6_ were initially dissolved in distilled water. A different mass fraction of ionic liquid (BmImPF_6_ or BmImTf) was thus added to the solution. The solution was then stirred overnight at 80 °C. The resulting solution was cast on a glass Petri dish and dried in an oven. Biopolymer electrolytes were eventually produced.

### 3.3. Characterizations of Polymer Electrolytes

Freshly prepared samples were subjected to ac-impedance spectroscopy for ionic conductivity measurements at ambient temperature. The thickness of the biopolymer electrolytes were measured using a digital micrometer screw gauge. The ionic conductivity of the biopolymer electrolytes was measured with the HIOKI 3532-50 LCR HiTESTER electrochemical impedance analyzer (Hioki, Japan) from 5–50 MHz at ambient temperature at a signal level of 10 mV. Biopolymer electrolytes were sandwiched between two stainless steel (SS) blocking electrodes.

### 3.4. Electrode Preparation

Activated carbon**-**based electrodes were prepared by a dip coating technique. The carbon slurry was prepared by mixing 80 wt% of activated carbon (Kuraray Chemical Co. Ltd., Osaka, Japan, particle size is 5–20 μm and surface area is 1800–2000 m^2^·g^−1^), 10 wt% of Super P and 10 wt% of poly(vinylidene fluoride) (PVdF) which acts as binder (molecular weight of 534,000 g·mol^−1^ from Aldrich) in 1-methyl-2-pyrrolidone (Purity ≥99.5% from Merck, Germany). This slurry was stirred thoroughly for several hours at ambient temperature until a homogenous slurry with a smooth surface was obtained. The weight of electrode materials was determined including the binder and super P. The aluminum electrode mesh was thus coated with the prepared carbon slurry using a dip coater. The coated electrodes were dried in an oven at 70 °C for the drying process.

### 3.5. Supercapacitor Fabrication

The supercapacitor was fabricated by sandwiching a biopolymer electrolyte between two symmetrical activated carbon-based electrodes. The supercapacitor was eventually placed in a cell kit for further electrochemical characterization.

### 3.6. Supercapacitor Characterization

The fabricated supercapacitor was subsequently subjected to linear sweep voltammetry (LSV), cyclic voltammetry (CV) and galvanostatic charge-discharge (GCD) testers. The supercapacitor containing BmImPF_6_ was designated as “Hexafluorophosphate system”, whereas the “Triflate system” is the name for the supercapacitor using BmImTf.

#### 3.6.1. Linear Sweep Voltammetry (LSV)

The electrochemical stability range was determined by a CHI600D electrochemical analyzer. This cell was analyzed at a scan rate of 5 mV·s^−1^ with the configuration of stainless steel (SS)/biopolymer electrolyte/SS in the potential range of ±3 V. The sample interval was 0.001 V with 2 s as the rest time before the measurement.

#### 3.6.2. Cyclic Voltammetry (CV)

The CHI600D electrochemical analyzer was also used to study the CV profile of the supercapacitor. Prior to the measurement, the cell was initially rested for 2 s to reach the equilibrium state. The supercapacitor was then analyzed at 10 mV·s^−1^ scan rate in the potential range between 0 and +1 V with sample interval of 0.001 V. The specific capacitance (*C*_sp_) of the supercapacitor was calculated using the following equation [7,18]:

$$C_{\mathrm{sp}}=\frac{i}{sm}$$ (1)

where *i* is the average anodic-cathodic current (A); *s* is the potential scan rate (V·s^−1^) and *m* is the average mass of active materials (including the binder and super P). The average mass of the electrode materials for both systems is around 0.02 g.

#### 3.6.3. Electrochemical Impedance Spectroscopy (EIS)

The impedance of the EDLC was probed by a HIOKI 3522-50 LCR HiTESTER impedance analyzer at room temperature with a bias voltage of 10 mV. The EIS measurements were done in the frequency range from 10 to 100 kHz. The capacitances C were determined from the impedance data at a frequency of 10 mHz using the following equation:

$$C=-\frac{1}{\omega Z"}=-\frac{1}{2\pi f\times Z"}$$ (2)

where ω is angular frequency, which is represented by 2π*f* and *Z*’’ is the imaginary part of the complex impedance (Z). The specific capacitance (*C*_sp_) of the EDLC was calculated by dividing the capacitance by the average weight of active materials. The average weight of electrode materials for both systems is 0.015 g.

#### 3.6.4. Galvanostatic Charge-Discharge Performance (GCD)

The charge-discharge study was accomplished using a Neware battery cycler. The supercapacitor was charged and discharged at current of 1 mA. The supercapacitor is allowed to rest for 30 min prior to the measurement. The specific discharge capacitance (*C*_sp_) was obtained from charge-discharge curves, according to the following relation [18]:

Csp=Im(dVdt) (3)

where *I* is the applied current (A); *m* is the average mass of electrode materials (including the binder and super P); d*V* represents the potential change of the discharging process excluding the internal resistance drop occurring at the beginning of the cell discharge; and d*t* is the time interval of the discharging process. The d*V*/d*t* is determined from the slope of the discharge curve. The average mass of electrodes in the hexafluorophosphate system is around 0.045 g, whereas for the triflate system, the average mass of electrodes is 0.015 g.

Energy density (*E*, W·h·kg^−1^), power density (*P*, W·kg^−1^) and Coulombic efficiency (η, %) were evaluated from the equations below [5]:

$$E=\frac{C_{\mathrm{sp}}\times {\left(dV\right)}^{2}}{2}\times \frac{1000}{3600}$$ (4)

$$E=\frac{C_{\mathrm{sp}}\times {\left(dV\right)}^{2}}{2}\times \frac{1000}{3600}$$ (5)

$$\eta =\frac{t_{d}}{t_{c}}\times 100\%$$ (6)

where *t*_d_ and *t*_c_ are the discharging time and charging time, respectively.

## 4. Conclusions

Two different types of ionic liquid-based biopolymer electrolytes were prepared by a solution casting method. The triflate based biopolymer electrolytes exhibit higher ionic conductivity than the hexafluorophosphate based biopolymer electrolytes due to the delocalization process of the triflate anions. The supercapacitor containing the triflate based biopolymer electrolyte also illustrates better electrochemical performances compared to that of the hexafluorophosphate based biopolymer electrolyte. The potential window range and capacitance of the supercapacitor are improved by adding a triflate based ionic liquid. No Faradic process was observed in CV and GCD studies. The supercapacitor based on the triflate polymer system possesses excellent electrochemical stability upon charging and discharging for 500 cycles.

## Figures and Tables

**Figure 9.**
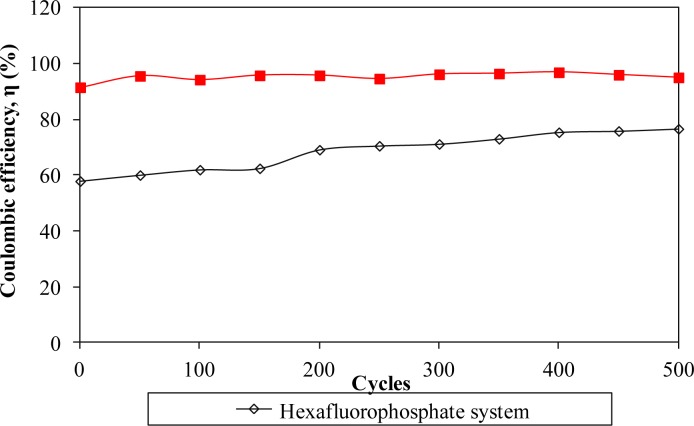
Coulombic efficiency of different ionic liquid-based biopolymer electrolyte systems over 500 cycles.

**Figure 10.**
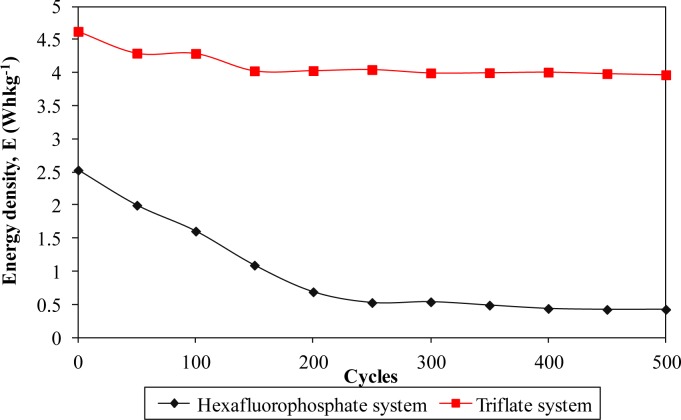
Energy density of different ionic liquid-based biopolymer electrolyte systems over 500 cycles.
